# BIN1 regulation of brain region‐specific tau pathogenesis and propagation

**DOI:** 10.1002/alz.087067

**Published:** 2025-01-03

**Authors:** Moorthi Ponnusamy, Shuai Wang, Melike Yuksel, Om Patel, Mitchell Hansen, Lisa Collier, Gopal Thinakaran

**Affiliations:** ^1^ Byrd Alzheimer’s Center & Research Institute, Tampa, FL USA

## Abstract

**Background:**

*BIN1*, the second strongest GWAS risk factor for late‐onset Alzheimer’s disease (AD), encodes a nucleocytoplasmic adaptor protein that plays many roles in multiple tissue and cell types. It is known that BIN1 can directly bind to tau *in vitro*, and neuronal BIN1 expression decreases in patients with AD. Accumulation of intracellular hyperphosphorylated tau is a hallmark pathogenic feature of AD and related tauopathies. Neuronal BIN1 localizes to presynaptic terminals and influences excitatory synaptic transmission, however, the molecular events underpinning neuronal BIN1 function in disease progression i*n vivo* has remained unclear.

**Method:**

To mimic the decrease in neuronal BIN1 expression in Alzheimer’s patients, we recently generated and characterized tau pathology in novel conditional *Bin1* knock‐out (tau P301S transgenic mice lacking BIN1 expression in the forebrain excitatory neurons; PS19:*Bin1*‐cKO) and examined tau pathogenesis. In order to establish a direct connection between neuronal BIN1 and the degree of neurofibrillary tangle pathology, we injected tau seeds into the brains of PS19:*Bin1*‐cKO mice and investigated tau propagation and spreading.

**Results:**

We observed that the loss of excitatory neuronal BIN1 expression attenuates tau accumulation and neurodegeneration selectively in the hippocampus, entorhinal/piriform cortex, and amygdala. Furthermore, the knock‐out mice had significantly reduced neuroinflammation, preservation of hippocampal synapses, and complex transcriptomic changes in the neurons and glial cells. In addition, we observed reduced brain‐derived tau seed propagation from the site of injection in the hippocampus into connected cortical regions. Instead, there is an accumulation of Ser202/Thr205 phosphorylated tau and MC1^+^ tau in hippocampal CA1 pyramidal neurons. Thus, our findings reveal an interesting region‐specificity in neuronal BIN1 regulation of tau pathogenesis and propagation.

**Conclusion:**

Overall, our findings reveal that excitatory neuronal BIN1 promotes region‐specific tau pathogenesis and tau propagation through neuroanatomically connected brain regions. These findings add to our understanding of *in vivo* BIN1 function in the context of tau pathogenesis, revealing cell‐autonomous and non‐cell‐autonomous mechanisms involved in BIN1 modulation of tau burden in AD.

